# Glutathione-Mediated Neuroprotective Effect of Purine Derivatives

**DOI:** 10.3390/ijms241713067

**Published:** 2023-08-22

**Authors:** Nobuko Matsumura, Koji Aoyama

**Affiliations:** Department of Pharmacology, Teikyo University School of Medicine, 2-11-1 Kaga, Itabashi, Tokyo 173-8605, Japan; nmatsumu@med.teikyo-u.ac.jp

**Keywords:** glutathione, purine derivatives, caffeine, uric acid, excitatory amino acid carrier protein 1, cysteine, Alzheimer disease, Parkinson’s disease

## Abstract

Numerous basic studies have reported on the neuroprotective properties of several purine derivatives such as caffeine and uric acid (UA). Epidemiological studies have also shown the inverse association of appropriate caffeine intake or serum urate levels with neurodegenerative diseases such as Alzheimer disease (AD) and Parkinson’s disease (PD). The well-established neuroprotective mechanisms of caffeine and UA involve adenosine A_2A_ receptor antagonism and antioxidant activity, respectively. Our recent study found that another purine derivative, paraxanthine, has neuroprotective effects similar to those of caffeine and UA. These purine derivatives can promote neuronal cysteine uptake through excitatory amino acid carrier protein 1 (EAAC1) to increase neuronal glutathione (GSH) levels in the brain. This review summarizes the GSH-mediated neuroprotective effects of purine derivatives. Considering the fact that GSH depletion is a manifestation in the brains of AD and PD patients, administration of purine derivatives may be a new therapeutic approach to prevent or delay the onset of these neurodegenerative diseases.

## 1. Introduction

A variety of purine derivatives are produced from purine nucleotides (i.e., adenine nucleotide and guanine nucleotide) by metabolic processes occurring mainly in the liver. For instance, adenine nucleotides are metabolized to produce ATP, ADP, AMP, adenosine, inosine, hypoxanthine, and xanthine, and finally uric acid (UA). Caffeine (1,3,7-trimethylxanthine), a naturally occurring purine derivative, is metabolized into several dimethylxanthines including paraxanthine, theophylline, and theobromine, and finally into UA derivatives.

Each purine derivative has a particular physiological activity, and several purine derivatives such as adenosine, guanosine, caffeine, paraxanthine, theophylline, theobromine, and UA have been shown to possess neuroprotective activities [1,2,3,4,5,6,7,8,9]. Caffeine has an adenosine A_2A_ receptor (A_2A_AR) antagonizing activity that is involved in neuroprotection [5]. The neuroprotective activity of UA is attributed to its antioxidative activity [10,11]. The neuroprotective activities of both caffeine and UA are supported by several epidemiological studies, which show negative correlations of dietary caffeine intake and serum UA levels with the onset of neurodegenerative diseases such as Alzheimer disease (AD) [12,13,14,15] and Parkinson’s disease (PD) [16,17,18,19]. Recently, we have shown that caffeine and its metabolites, paraxanthine and UA, have neuroprotective activities, enhance cysteine (Cys) uptake, and increase intracellular glutathione (GSH) levels [20,21]. GSH is an intracellular antioxidant tripeptide molecule (glutamylcysteinylglycine), which plays an important role in neuronal survival under oxidative stress in the central nervous system (CNS) [22,23]. Therefore, it is likely that the increase in GSH levels is related to the neuroprotective activities of purines such as caffeine, paraxanthine, and UA [20,21]. 

Increasing GSH levels is a neuroprotective mechanism that increases antioxidant activity in neurons. It is widely accepted that neuroprotective processes can be classified into three major mechanisms: (i) suppressing excessive neuronal stimulation, (ii) maintaining antioxidant activity in neurons, and (iii) detoxification of xenobiotics (Table 1). Intracellular GSH is directly or indirectly involved in all four of the intracellular antioxidizing pathways, i.e., Table 1 (ii, 1–4): intracellular GSH synthesis, Cys uptake, GSH supply from astrocytes, and antioxidant supply.

**Figure 1:**
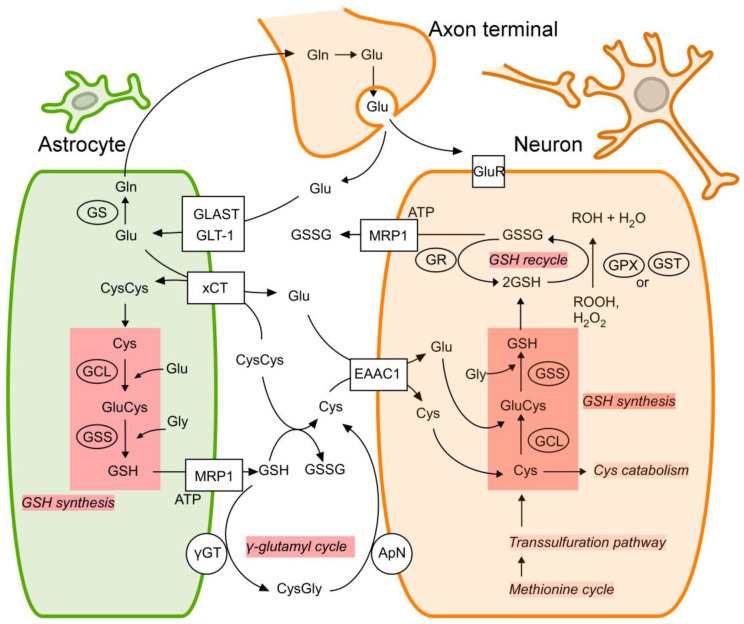
Regulations of GSH levels in neurons and astrocytes. In both neurons and astrocytes, GSH is synthesized from glutamate (Glu), cysteine (Cys), and glycine (Gly) by two-step enzymatic reactions catalyzed by GCL and GSS. Although Cys is synthesized through the methionine cycle and the transsulfuration pathway, Cys uptake via EAAC1 is the main mechanism supplying Cys to produce intracellular GSH in neurons. In astrocytes, cystine (cysteine disulfide, CysCys) is taken up via xCT and rapidly converted to Cys in intracellular space. In neurons, glutathione disulfide (GSSG), the oxidized form of GSH, is released via MRP1. Astrocytic GSH is released into extracellular space via MRP1. GSSG is recycled to GSH by GSH reductase (GR, Gene ID: 2936), and the released GSH is converted to Cys through the γ-glutamyl cycle. Neurons take up extracellular Cys and use it to synthesize GSH. Other abbreviations: ApN, aminopeptidase N; GLAST, glutamate-aspartate transporter (Gene ID: 6507); Gln, glutamine; GLT-1, glutamate transporter 1 (Gene ID: 6506); GluCys, glutamylcysteine; GluR, glutamate transporter; GPx, glutathione peroxidase (Gene ID: 2876); GS, glutamine synthetase; GST, glutathione S-transferase; CysGly, cysteinylglycine; γ-GT, γ-glutamyl transpeptidase.

Figure 1 summarizes the regulation of GSH synthesis in neurons and astrocytes. GSH is synthesized from glutamate (Glu), Cys, and glycine (Gly) by two-step enzymatic reactions [32,49]. The first step of GSH synthesis is catalyzed by γ-glutamyl cysteine ligase (GCL), which is composed of a GCL catalytic subunit (GCLC, Gene ID: 2729) and a GCL modifier subunit (GCLM, Gene ID: 2730) [30,31,32]. The second step of GSH synthesis is catalyzed by GSH synthetase (GSS, Gene ID: 2937) [33]. The GSH synthesis process is preceded by Cys uptake, which is mediated by excitatory amino acid carrier protein 1 (EAAC1, Gene ID: 6505) in neurons [35,36]. Cys uptake into neurons is a major pathway that supplies material for GSH production. Transporting Cys is mainly mediated by a neuron-specific Cys transporter, EAAC1 [34,35,50]. 

The intracellular presence of the antioxidants such as ascorbate, UA, and α-tocopherol help to preserve neuronal GSH levels by doing the work of protecting cells against oxidative stress through other means [11,40,41,42,43]. Neuronal GSH levels are also affected by GSH production in the astrocytes that surround and support the neurons [28,37,51]. Astrocytes release GSH via multidrug resistance protein 1 (MRP1, Gene ID: 4363); the GSH is degraded extracellularly and then taken up by neurons to be reconstructed [39,52]. This GSH release is promoted by enhancing GSH synthesis in astrocytes, which is mainly regulated by GCL, GSS, and cystine (cysteine disulfide) uptake via the cystine/glutamate antiporter, system xc^−^ (xCT, Gene ID: 23657) [38] (Figure 1). 

Several purine derivatives are found to be neuroprotective, and they increase intracellular GSH levels not only in neurons but also in astrocytes. Thus, the regulation of GSH levels is likely integral to the neuroprotective activity of purines in the CNS. We present here the relation of purine derivatives with neuroprotection, especially in terms of GSH synthesis (in Section 3.3). 

Whereas increased GSH is known to be neuroprotective, reduced brain GSH levels have been reported to precede the pathologic hallmarks of AD such as amyloid oligomerization and plaque formation in AD model mice [53]. In a clinical study, GSH depletion was considered an early event in the progression of PD [54]. Thus, promoting intracellular GSH synthesis prior to symptomatology may halt the progression of neurodegenerative diseases such as AD and PD. Basic research on the effect of purine derivatives on increasing GSH levels is valuable for developing novel disease-modifying drugs for the treatment of neurodegenerative diseases. 

In this review, we summarize the role of purine derivatives in enhancing neuroprotective activities and alleviating neurodegenerative insults. In the second section, we describe recent epidemiological studies on the relationship of both caffeine and UA to the onset of neurodegenerative diseases. Then, in the final section, we present the neuroprotective functions of purine derivatives especially in terms of GSH synthesis. 

## 2. Epidemiological Studies of the Relation between Caffeine or UA and Lower Risks of Neurodegenerative Diseases

Aging societies such as that in Japan face serious problems of age-related neurodegenerative disease such as AD and PD and especially the cognitive decline that accompanies these diseases. No effective curative or prophylactic treatment for the development of AD or PD has so far been clinically developed. Genetic backgrounds are involved in the onset and progression of familial AD and PD [55,56], while several environmental factors that promote or attenuate the onset of sporadic AD [57] and PD [58,59] have been identified in epidemiological studies. Previous studies have suggested the involvement of factors such as caffeine intake and serum UA levels in modulating the incidence or progression of AD and PD. Serum caffeine concentrations in PD patients and matched healthy control are about 2.4 µM and 7.9 µM, respectively [60]. UA concentration in PD patients and matched healthy control is about 274 µM and 286 µM, respectively [19]. A higher serum UA (≥271 µM) is associated with lower risk for AD compared to lower serum UA (≤210 µM) [15]. The neuroprotective activities of both caffeine and UA have been confirmed by preclinical studies using animal models. These studies suggest that the neuroprotective mechanisms of caffeine and UA are respectively involved in A_2A_AR antagonism (*K_b_* = 12.3 µM, Table 2) [3,61,62] and in antioxidant activity (200 to 500 µM UA) [63,64], namely, with upregulation of the signaling pathway that produces antioxidative molecules in cells. 

### 2.1. Relation between Caffeine Intake and Lower Risk of Neurodegenerative Diseases

Since the 1980s, numerous epidemiological studies have shown a correlation between coffee/caffeine intake and lowering the risk of neurodegenerative diseases such as AD and PD. A negative correlation was found between caffeine intake and the incidence or progression of AD [12,13,16]. In one study, caffeine intake was correlated with a lower risk of cognitive decline in women, but not significantly so in men [12]. To clarify whether the coffee-induced effect could be attributed to molecules other than caffeine, Dong et al. [13] reported the contribution of caffeine to reducing the risk of cognitive decline in elder adults by comparing five different groups, including those who did not consume coffee, and those who consumed coffee, caffeinated coffee, decaffeinated coffee, and caffeine from coffee. The results showed that cognitive performance was correlated with the intake of coffee, caffeinated coffee, and caffeine from coffee, but not decaffeinated coffee. Thus, these findings suggest that intake of caffeine, rather than other components of coffee, significantly lowers the risk of cognitive decline of AD patients.

Coffee/caffeine intake has also shown correlations with better cognitive performance in PD patients [16,17,19,70,71,72,73,74,75,76,77,78,79]. Although the correlation between coffee intake and the lower risk of PD was not significant in early studies [70,71], subsequent larger case-control studies demonstrated the relation of a lower risk of PD to coffee/caffeine intake [16,17,19,72,73,74,75,76,77,78,79]. Ross et al. [17] showed that coffee/caffeine intake was correlated with a low incidence of PD, whereas tobacco use (smoking) and administration of other nutrients contained in coffee did not lower the risk of PD.

More than 90% of caffeine clearance is mediated by cytochrome P450 family 1 subfamily A member 2 (CYP1A2, Gene ID: 1544) [80]. A polymorphic variant of CYP1A2 (–163 C > A) (GenBank accession number AF253322) confers higher CYP1A2 inducibility and higher individual caffeine metabolic activity [81]. Recently, Tan et al. [82] examined the correlation of caffeine intake with PD risk in terms of caffeine metabolism: in their case-control study, there was no difference in the relationship of caffeine intake to low risk of PD between fast and slow caffeine metabolizer genotypes. Even after normalizing caffeine absorption and metabolism, reduced salivary caffeine levels in PD patients correlates with PD progression [83]. These results further support the neuroprotective activities of both caffeine and its major metabolite, paraxanthine, found in animal studies [6,21,84]. However, it remains unclear whether paraxanthine alone is correlated with a lower risk of PD. 

### 2.2. Relation between Serum UA and Risk of Neurodegenerative Diseases

High serum UA levels cause some critical diseases such as gout, cardiovascular disease, hypertension, and renal disease [85], while it has neuroprotective effects in PD and AD animal models, because UA has antioxidant activity. Several clinical studies have demonstrated the neuroprotective effect of high serum UA levels on neurodegenerative diseases such as PD and AD [19,86].

Although some conflicting results have been reported [87], a significant correlation between serum UA levels and a low risk of PD have been shown by many studies [19,88,89,90,91,92]. Some of the studies identify a difference in the UA/PD relationship between men and women [18,93]. In one study, higher plasma UA levels correlated with lower risk of PD in men but not in women [93]. Cortese et al. [18] also demonstrated that higher serum UA levels are related to lower risk of PD in men. Although the relation is weaker in women, the protective effect of serum UA is significantly increased in aged women (above 70 years), whose UA levels are higher than those in premenopausal women. These studies further support the correlation between serum UA levels and lower risk of PD.

A strong correlation of plasma antioxidant levels with both mild cognitive impairment and AD has been observed [14,86]. Furthermore, it has been shown that serum UA levels are correlated with lower risks of dementia, AD, and vascular dementia [15]. Interestingly, in participants without dementia, there was a correlation between higher serum UA levels and better cognitive performance in life; however, in participants with dementia, there was a correlation of high serum UA levels with declines in cognitive performance and manifestation of brain atrophy [94]. 

As mentioned above, some studies have supported the potential role of hyperuricemia to prevent AD, while other studies have reported conflicting results or have failed to demonstrate any significant association [95,96]. Although the results of these clinical studies have been inconsistent, a recent meta-analysis supported the correlation between serum UA levels and scores on the Mini-Mental State Examination in patients with PD-related dementia [14,15,94,97].

By treatment with urate-lowering agents, patients with gout maintain lower serum UA levels over long periods of time. One might think that low serum UA levels would impair neurons by increasing oxidative stress in the brain. However, use of urate-lowering drugs (allopurinol, febuxostat) is not correlated with any increase in the risk of dementia [95]. Treatment with pegloticase, a PEGylated urate oxidase (uricase), reduced mean serum UA levels more than 90% (from 10.8 to 0.9 mg/dL = 642 to 53 μM); however, there was no significant increase in biomarkers of oxidative stress, and the levels of oxidative markers did not correlate with serum UA levels [98]. Thus, drug-controlled low serum UA levels do not appear to affect the development of dementia or oxidative stress. The lack of correlation between low levels of serum UA and the development of dementia may be due to the absence of elevated oxidative stress levels. Indeed, it is not yet fully understood whether UA acts as an antioxidant in blood [11,99] and how serum UA provides neuroprotection in the CNS.

## 3. Neuroprotective Activities of Purines

### 3.1. Regulations of UA Levels in Blood and the Brain

**Figure 2:**
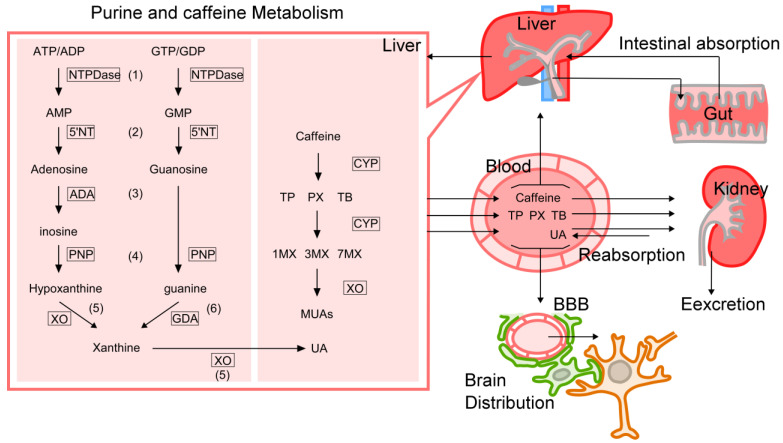
Purine metabolism in peripheral tissues. (1) ATP is converted to ADP and AMP by nucleoside triphosphate diphospho-hydrolases (NTPDases; CD39), (2) AMP is converted to adenosine by 5′-nucleotidase (5′NT; CD73), (3) adenosine is converted to inosine by adenosine deaminase (ADA), (4) inosine is converted to hypoxanthine by purine nucleoside phosphorylase (PNP), and (5) xanthine oxidase (XO) catalyzes the conversion of hypoxanthine to xanthine and finally to UA. GTP is converted to GDP and GMP by NTPDases. GMP is converted to guanosine by 5′NT, guanosine is transformed into guanine by PNP. Guanine is converted to xanthine by guanine deaminase (GDA, Gene ID: 9615). Caffeine (1,3,7-trimethylxanthine) is demethylated to paraxanthine (PX), theobromine (TB), and theophylline (TP) by cytochrome P450 (CYP). PX, TB, and TP can be further demethylated by CYP to monomethylxanthines such as 1-methylxanthine (1MX), 3MX, and 7MX. The 8-hydroxylation of MXs to form corresponding methyl UAs (MUAs) is mainly catalyzed by XO.

Serum UA levels are maintained by food digestion, purine synthesis, metabolism, and purine excretion into urine. The net excretion of UA in urine is determined by the balance between UA re-absorption and secretion within the proximal tubule, and each process is mediated by its specific transporter (Figure 2). Since uricase activity is absent in primates, UA is the end metabolite of purines in humans. Therefore, serum UA levels in humans are higher than in other mammals. Abnormally high UA levels are related to the onset of diseases such as gout, cardiovascular disease, hypertension, and renal disease [85] because UA behaves as a pro-oxidant under certain circumstances: for example, in the presence of transition metals in the microenvironment [100]. However, higher UA levels in serum and cerebrospinal fluid have been effective in protecting neurons from oxidative stress in animal studies [8,9,101] and have been related to a lower onset of AD and PD in clinical studies (as described in Section 2).

**Figure 3:**
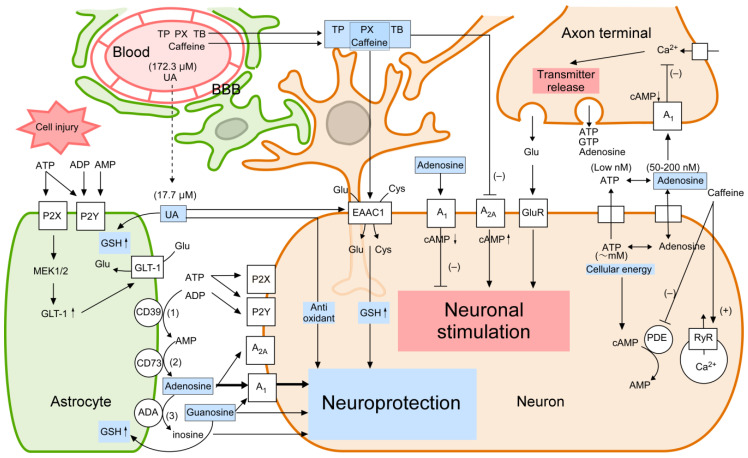
Purine metabolism and neuroprotection. (1) ATP is converted to ADP and AMP by CD39, (2) AMP is converted to adenosine by CD73, (3) adenosine is converted to inosine by ADA. The neuroprotective mechanism of UA is its antioxidant activity, while that of caffeine is A_2A_AR receptor antagonism. Adenosine reduces neuronal excitability and firing rate via A_1_AR stimulation. The mechanism of guanosine induced neuroprotection involve A_1_AR agonism. Upregulation of GSH levels is one of the neuroprotective mechanisms of UA, caffeine, PX, and guanosine. Abbreviations: cAMP, cyclic AMP; MEK, extracellular-signal regulated kinase kinase; P2X, ionotropic purinergic receptor; P2Y, metabotropic purinergic receptor.

UA is also produced during ATP metabolism through the following steps [102]: (1) ATP is converted to ADP and AMP by nucleoside triphosphate diphospho-hydrolases (NTPDases; CD39, Gene ID: 953) [103], (2) AMP is converted to adenosine by 5′-nucleotidase (5′NT; CD73, Gene ID: 4907) [104], (3) adenosine is converted to inosine by adenosine deaminase (ADA, Gene ID: 100), (4) inosine is converted to hypoxanthine by purine nucleoside phosphorylase (PNP, Gene ID: 4860), and (5) xanthine oxidase (XO, Gene ID: 7498) catalyzes the conversion of hypoxanthine to xanthine and finally to UA [105,106] (Figure 2). In mammals, XO is abundant in the liver, intestine, and mammary gland whereas it is scarce in the heart, muscle, and brain [105]. In immunolocalization studies, XO protein is located in mammary epithelial cells and in the capillary endothelial cells of almost all tissues except the brain and testis in bovines. The human brain lacks XO, and the XO activity is absent from neurons, astrocytes, epithelial cells, endothelial cells, and capillary endothelial cells in the brain [105]. Importantly, because UA production mainly occurs in the liver and not in the brain, serum UA can be supplied to the brain only by passing through the blood–brain barrier (BBB) (Figure 3).

In 1981, Granger et al. and McCord et al. [107,108] hypothesized that XO-generated reactive oxygen species (ROS) cause ischemic reperfusion injury in bowel and cardiac tissue. AMP catabolism to hypoxanthine occurs under hypoxic conditions in the ischemic process, while in the reperfusion process, XO and O_2_ mediate hypoxanthine oxidation to form xanthine and the end metabolite, UA, concomitant with super oxide anions (O_2_^−^). Therefore, the lack of XO in the brain may protect neurons from such oxidative stress. In vivo preclinical study has shown that UA protects hippocampal neurons after ischemia reperfusion in rats [109].

In terms of the mechanism by which UA in blood is involved in neuroprotection in the brain, Amaro et al. [110] argued that the main target of UA is endothelial cells, because UA is practically unable to pass through the BBB. The protection of endothelial cells from ischemic injury leads to the survival of the whole neurovascular unit. When the BBB is impaired, UA passes through it. In fact, UA levels in cerebrospinal fluid correlate positively with serum UA levels, especially when the BBB is impaired [96]. The neuroprotective activity of UA has been reported in 6-hydroxydopamine (6-OHDA) lesioned PD model rats [8], as in ischemia-injured rats [9,109]. Although it is not clear how UA crosses the BBB and how it acts on the neurons, these results support the notion that systemic administration of UA might induce neuroprotective activity in the CNS. 

### 3.2. Regulation of Purine Metabolism in the Brain 

Other than nucleic acids, the typical intracellular purine derivative is ATP, which acts as an energy source for driving neuronal activity. Huge amounts of ATP (on the order of several mM) are produced in nervous tissues to maintain energy for Na^+^/K^+^ ATPase and synaptic transmission [111,112,113]. In contrast, extracellular ATP (at a concentration of low nM) regulates cellular signaling via P2-purinergic receptors such as P2Y and P2X [114,115,116] (Figure 3). ATP is released from cells under physiological conditions [117] and is also released from damaged cells [118,119,120] (Figure 3).

ATP is converted to ADP, AMP, adenosine, inosine, hypoxanthine, and xanthine and finally yields UA in the peripheral tissues (Figure 2). These purine derivatives have a variety of physiological activities by modulating adenosine receptors in the brain. Methylxanthines (MX) such as caffeine, paraxanthine, theophylline, and theobromine can be converted to UA by both demethylation and oxidation processes (Figure 2). 

Adenosine is formed from AMP intracellularly in the CNS. Under physiological conditions, ATP and adenosine can be released from presynaptic neurons. Adenosine is also produced in extracellular space from AMP derived from released ATP and cyclic AMP (cAMP) [121,122,123]. In addition, astrocytes in the brain use ATP to regulate the extracellular concentration of purines, the metabolism of which is catalyzed by ecto-enzymes on cell membranes [122,124]. ATP is rapidly converted to ADP, AMP, adenosine, and inosine in the extracellular space [117,120,125] (Figure 3). ATP is decomposed into ADP and AMP by CD39 [103], AMP is decomposed into adenosine by CD73 [126], and adenosine is decomposed into inosine by ADA [127]. Adenosine stimulates adenosine A_1_ receptor (A_1_AR) and A_2A_AR (*EC*_50_ values for A_1_AR and A_2A_AR are 0.31 µM and 0.73 µM, respectively, Table 2). Trauma and ischemia induce the expression of CD39 and CD73 in astrocytes and increase extracellular adenosine derived from ATP metabolism [126,128] (Figure 3). Clearly, a variety of purine derivatives are produced from metabolic processes of purine nucleotides, and at least some of them seem to have neuroprotective activity in vitro and in vivo. 

### 3.3. Neuroprotective Mechanisms of Purines

As described in the Introduction and illustrated in Table 1, neuroprotective processes can be classified into three major categories (Table 1): (i) suppressing excessive neuronal stimulation by neurotransmitter antagonism, (ii) maintaining antioxidant activity in neurons, which limits cellular oxidative stress caused by electrophile molecules such as ROS, and (iii) detoxifying xenobiotics such as nucleophile molecules and abnormally aggregated proteins such as α-synuclein and amyloid β peptide. Known neuroprotective activities induced by purine derivatives have been classified in categories (i) and (ii) above, but category (iii)-mediated neuroprotective effects of purine derivatives have been unclear.

#### 3.3.1. Neuroprotection by Antioxidative Activity

UA has antioxidant activity [11,129,130] that leads to an established neuroprotective property, while xanthine structure has no such activity [64]. Structure-activity relationships for purine derivatives indicate that the 8-one substituent in the chemical structure of UA plays an important role in its antioxidant properties [63,129]. It has been suggested that UA may neutralize reactive oxygen species produced via a Fenton-type chemical reaction in cells. However, UA levels in cerebrospinal fluid (17.7 µM) are lower than those in plasma (172.3 µM) [96] (Figure 3), suggesting that UA might have an antioxidant effect in blood. 

UA both acts as a scavenger of ROS and peroxynitrite [99] and prevents iron-mediated ascorbate oxidation [131]. In vitro, UA has an antioxidant activity similar to that of ascorbate, but humans have much higher levels of UA than ascorbate, due to their loss of uricase function during the course of evolution. However, low serum UA levels do not increase oxidative stress in blood [98]. In fact, UA is a less effective antioxidant than ascorbate in human blood [10,11]. This is because of UA’s alternative role as an iron chelator. Therefore, UA inhibits iron-catalyzed oxidation of ascorbate and stabilizes ascorbate levels in serum [132]. Antioxidant activity is observed at UA concentrations of more than 200 µM [10,64]; however, a much lower concentration of UA (10 µM) was shown to increase Cys uptake through the Cys transporter, resulting in GSH synthesis in hippocampal slices [20]. Thus, UA may contribute neuroprotection not only by antioxidant activity but also by promoting GSH production in neurons.

The neuroprotective activities of UA and caffeine have been confirmed by several preclinical studies. The well-established neuroprotective mechanism of UA is its antioxidant activity (10 to 100 µM), while that of caffeine is A_2A_AR receptor antagonism (*IC*_50_ = 107 µM, Table 2). Furthermore, UA and caffeine have other neuroprotective mechanisms in which GSH levels are increased by upregulating expressions of antioxidant-related proteins (Figure 3 and Table 3). UA appears to increase GSH levels by enhancing expression of nuclear factor erythroid-2-related factor 2 (Nrf2)-responsive genes, including GCLC, heme oxygenase-1 (HO-1), and NAD(P)H quinone oxidoreductase 1 (NQO1), and the resultant GSH increase provides antioxidant activity (Table 3).

There are numerous examples of UA’s direct neuroprotective roles. UA protects PC12 cells from 6-OHDA-induced cell injury by increasing levels of both GSH and superoxide dismutase (SOD) protein [133]. UA treatment enhances SOD activity, increases GSH levels, and reduces oxidative products of malondialdehyde (MDA) in a 6-OHDA-induced PD model rat [8]. In 1-methyl-4-phenyl-1,2,3,6-tetrahydropyridine (MPTP)-treated PD model mice, UA improves behavioral performance and cognition, and UA also prevents the cell death of dopaminergic neurons, which is induced by modulation of neuroinflammation and oxidative stress [101]. UA treatment enhances Nrf2-responsive gene expression, including GCLC, HO-1, and NQO1, increases SOD, catalase (CAT), and GSH levels, and reduces MDA in the substantia nigra [101] (Table 3). 

UA also has an indirect neuroprotective activity by increasing GSH levels in astrocytes. UA treatment activates Nrf2 and leads to upregulation of gene expression of the GCLM, which enhances GSH production in astrocytes [37] (Table 3). This increase in astrocytic GSH levels can enhance GSH release into extracellular space, and the released GSH can be converted to Cys, which is taken up by neurons [37].

Nrf2 activation is another indirect mechanism by which purine derivatives provide neuroprotection. Nrf2 activation was observed when caffeine was treated as a neuroprotective agent in lipopolysaccharide (LPS)-induced oxidative stress model mice [134] and cadmium-induced cognitive deficit model mice [135] (Table 3). Nrf2 activators such as tert-butylhydroquinone (t-BHQ) and L-sulforaphane can induce EAAC1 expression by the Nrf2/ARE pathway in C6 glioma cells [141]. In vivo treatment of t-BHQ upregulates EAAC1 expression by Nrf2 activation and increases neuronal GSH levels in the mouse striatum [141]. T-BHQ also upregulates xCT expression in astrocytes in vitro [142]. Although caffeine-induced EAAC1 expression via A_2A_AR antagonism is observed in the developing retina [143], it is still unclear whether caffeine-induced Nrf2 activation upregulates EAAC1 or xCT and leads to increasing GSH synthesis. 

Our previous study showed a novel mechanism by which treatment with caffeine (10 to 100 µM) and UA (1 to 10 µM) provided neuroprotection by increasing GSH levels in the brain. The upregulation of GSH synthesis is mediated by increasing Cys uptake via EAAC1 in hippocampal slices [20]. In addition, paraxanthine (1,7-dimethylxanthine), a major metabolite of caffeine, increases Cys uptake in hippocampal slices whereas other purine metabolites (theophylline, theobromine, 1-MX, 3-MX, and 1,7-methyluricacid) do not [21]. Paraxanthine (10 to 100 µM) increases Cys uptake in human neuroblastoma SH-SY5Y cells via EAAC1 transport activity, and paraxanthine also exhibits neuroprotective activity in SH-SY5Y cells [21]. The upregulation of GSH levels is independent of both A_1_AR and A_2A_AR antagonisms, because an A_2A_AR antagonist, SCH58261, did not increase GSH levels in hippocampal slices [20], and because UA exhibits no activity on A_1_AR (*IC*_50_ > 5300 µM, Table 2) [68].

In astrocytes, guanosine upregulates GCL, glutamine synthetase (GS), and GSH reductase (GR), resulting in elevated GSH levels [136,137] (Table 3). The increase in GSH levels in astrocytes may protect neurons against oxidative stress by promoting GSH supply to neurons. Thus, GSH-regulated gene expression is one of the neuroprotective mechanisms of UA, caffeine, and guanosine alike. 

Finally, guanosine is a nucleotide metabolite that acts as an efficient neuromodulator in the brain, and its extracellular role has recently been clarified [4,137,144]. Extracellular guanosine is found to be neuroprotective and active in neurological regeneration in response to brain ischemia and trauma [4]. The mechanisms of guanosine-induced neuroprotection involve A_1_AR and A_2A_AR (of which it is a weak agonist [4]), Kir 4.1 potassium channels (Kir 4.1), and the excitatory amino acid transporter, glutamate transporter 1 (GLT-1). In neurons and astrocytes, guanosine activates pro-survival pathways such as Phosphoinositide 3-Kinase (PI3K), protein kinase B (Akt), and extracellular-signal regulated kinase kinase (MEK)/extracellular-signal regulated kinase (ERK) via adenosine receptors [138,145]. Guanosine also exerts neuroprotective activities by upregulations of PI3K/Akt and MEK/ERK signals in rat cortical astrocytes [138], and upregulation of PI3K/Akt/glycogen synthase kinase 3 (GSK3) signal in rat hippocampus slice [139] (Table 3). In vivo treatment of guanosine treatment improves behavioral performance and reduces mitochondrial dysfunction in the penumbra area in ischemia model rat [146].

#### 3.3.2. Neuroprotection by Adenosine Receptor Modulation

Adenosine is another purine that has neuroprotective activities. Since the 1940s, adenosine has been used clinically for cardiac protection and vasodilation [147]. Adenosine exhibits both neurostimulative and neuroprotective activities. Its neuroprotective activity is typified as repression of excess neuronal activation (category (i) in Table 1). Extracellular adenosine at concentrations of 0.05 to 0.2 µM is sufficient to modulate synaptic functions [147]. The actions of adenosine in the brain are mediated by A_1_AR (*IC*_50_ = 0.070 µM), A_2A_AR (*IC*_50_ = 0.150 µM), adenosine A_2B_ receptor (A_2B_AR, Gene ID: 136) (*IC*_50_ = 15.4 µM), and adenosine A_3_ receptor (A_3_AR, Gene ID: 140) (*IC*_50_ = 6.5 µM), which are variously activated, depending on adenosine concentrations [67,148,149] (Table 2). The expression of subtypes of adenosine receptors varies according to cell type. A_1_AR is the most abundant in the brain. Adenosine reduces neuronal excitability and firing rate via A_1_AR stimulation [150,151,152]. A_1_AR stimulation by adenosine induces neuroprotection in cultured neurons and ischemia model of rats [2,140] (Figure 3, Table 3). In vivo studies have shown that neuroprotection was induced by A_1_AR stimulation in animal models of ischemia [152,153]. A_1_AR stimulation inhibits Ca^2+^ entry into the presynaptic terminal, resulting in a reduction in neurotransmitter release [154,155,156] and hyperpolarizes postsynaptic neurons [157,158]. In contrast, A_2A_AR stimulation in the brain induces neuronal excitability and synaptic transmission [147,159]. Specific A_2A_AR antagonists such as KW-6002 (istradefylline), which is xanthine-based compound, protect nigral dopaminergic cells from damage induced by 6-OHDA in rats or by MPTP in mice [160]. Genetic depletion of A_2A_AR lowered the dopaminergic neurotoxicity in PD model animals [62]. These results indicate neuroprotective activities of both A_1_AR agonism and A_2A_AR antagonism [3].

Caffeine is another purine that offers category (i) neuroprotection through the A_2A_AR receptor, as well as its previously described category (ii) role of increasing intracellular GSH levels via Cys uptake. Methylxanthines such as caffeine and theophylline are known for their bronchoprotective effects. The molecular mechanisms include adenosine receptor antagonism (Caffeine; *IC*_50_ = 107 µM) and phosphodiesterase inhibition (Caffeine; *IC*_50_ = 400 µM) [69] (Table 2). A 30 mg/kg intraperitoneal injection of caffeine provides neuroprotective effects (Table 3). Since the serum caffeine concentration is 116 µM 60 min after the injection [161], it is enough concentration to antagonize adenosine receptors but not to affect phosphodiesterase activity. Of course, caffeine also has motor stimulant, psychostimulant, arousal, anti-inflammatory, anti-oxidative, and neuroprotective effects [69,162]. A_1_AR antagonism is involved in the stimulus effects of caffeine [163]. Of these, the arousal effect of caffeine (15 mg/kg, i.p.) is dependent on the A_2A_AR antagonism [164], and the anti-inflammatory effect of caffeine (100 µM) is mediated by both A_1_AR and A_2A_AR [165,166]. The locomotor stimulatory effect of high doses of caffeine cannot be attributed to the modulation of either the A_1_AR or the A_2A_AR, suggesting that this effect is independent of AR activity. It is accepted that the neuroprotective activities of caffeine are mediated by A_2A_AR inhibition [3]. Caffeine, paraxanthine, or theophylline (10 or 30 mg/kg, i.p.) prevents neuronal cell loss in MPTP-treated PD model animals [5,6] (Table 3). In fat-enriched diet-induced cognitive deficits rats, chronic treatment with theobromine (30 mg/day) improved cognitive functions and amyloid-beta protein (Aβ) and interleukin-1β (IL-1β) levels in the brain [7] (Table 3). Chronic treatment with caffeine resulted in tolerance to its motor stimulation activity, whereas caffeine-induced neuroprotection does not diminish with exposure [167]. Mechanisms other than adenosine receptors may also be involved in the neuroprotective effects of caffeine.

#### 3.3.3. Neuroprotection by Other Mechanisms

There are a few other, less pervasive mechanisms responsible for neuroprotective activities of purines. UA promotes pro-survival pathways such as activating PI3K/Akt, and MEK/ERK in the brain. For example, UA treatment (200 µM UA) prevents dopamine (DA) neuron loss and behavioral deficits in 6-OHDA treated rat by recovery of Akt/GSK3β signaling [8] (Table 3). A PI3K inhibitor was shown to interfere with UA-induced neuroprotection and regulations on Akt/GSK3β signaling in 6-OHDA-treated SH-SY5Y cells [8]. Thus, PI3K/Akt/GSK3β signaling can be involved in the neuroprotective activities of UA.

Ya et al. [9] have shown that UA treatment (UA 16 mg/kg i.v.) reduces focal cerebral ischemic reperfusion-induced oxidative stress, preventing neuronal damage. The mechanisms underlying this neuroprotective activity of UA are the upregulations of both brain-derived neurotrophic factor (BDNF) and nerve growth factor (NGF) via the Nrf2 signaling pathway. 

Paraxanthine at a concentration of 800 µM has a neuroprotective activity for dopaminergic neurons [84]. Its neuroprotective mechanism is attributed to a cytosolic calcium release from the endoplasmic reticulum via the activation of ryanodine receptor channels. However, this activity of paraxanthine is not mediated by antagonizing adenosine receptors or by elevation of intracellular cAMP (phosphodiesterase inhibition). 

ATP itself has no neuroprotective activity corresponding to categories (i) and (ii) in Table 1. However, ATP increases the expression of the astrocyte glutamate transporter, GLT-1, by P2Y (ATP receptor) stimulation, which activates the ERK/nuclear factor kappa-light-chain-enhancer of activated B cells (NF-κB) signaling pathway. Since upregulation of GLT-1 in astrocyte promotes the removal of excess glutamate from extra-synaptic space, ATP may be involved in this category (i) neuroprotective mechanism. Thus, extracellular purines such as adenosine and ATP regulate cellular signaling via their specific receptors. 

As described above, the neuroprotective mechanisms of purine derivatives are mainly due to adenosine receptor modulation and upregulation of the synthesis of proteins that are involved in increasing antioxidative activity or activating pro-survival pathways in cells. Many receptor activities develop tolerance to their antagonists [167], i.e., caffeine-induced arousal, psychostimulant, and motor stimulant activities develop tolerance to caffeine, but the neuroprotective activity does not. In addition to antagonizing A_2A_AR, increasing GSH levels via Cys uptake may play an important role in the neuroprotective activities of purine derivatives. 

## 4. Conclusions

Several purine derivatives exhibit neuroprotective activity mediated by increasing neuronal GSH levels, which can be due to not only induction of GSH synthesis-related enzymes, but also the upregulation of GSH levels explained by promoting EAAC1-mediated Cys uptake, which is a commonly seen after treatment with caffeine, UA, or paraxanthine.

The upregulation of GSH levels may enhance antioxidative activity in neurons and appears to be effective in preventing the incidence and progression of neurodegenerative disease. Further studies to investigate how purine derivatives increase GSH levels by promoting EAAC1-mediated Cys uptake would provide valuable insights into neuroprotection by purine derivative treatment. These studies of purine derivative biochemistry could lead to novel preventions and treatments for the growing burden of neurodegenerative disease in aging societies worldwide. 

## Figures and Tables

**Table 1:** Neuroprotective mechanisms.

Mechanism of Neuroprotection	References
(i) Suppressing excessive neuronal stimulation	
1. Antagonizing excitatory amino acids	[24,25]
2. Inhibiting neurotransmitter release	[26,27]
3. Promoting neurotransmitter uptake and metabolism	[28,29]
(ii) Maintaining antioxidant activity in neurons	
1. GSH syntheses (GCL and GSS)	[30,31,32,33]
2. Cysteine uptake (EAAC1)	[34,35,36]
3. GSH supply from astrocyte (GCL, xCT, and MRP1)	[28,37,38,39]
4. Other antioxidants (ascorbate, UA, and α-tocopherol)	[11,40,41,42,43]
(iii) Detoxifying xenobiotics	
1. Induction of phase I enzymes	[44,45]
2. Induction of phase II enzymes	[46]
3. Exclusion via MRPs	[47,48]

Abbreviations: EAAC1, excitatory amino acid carrier protein 1; GCL, γ-glutamyl cysteine ligase; GSH, glutathione; GSS, GSH synthetase; MRP1, multidrug resistance protein 1; MRPs, multidrug resistance proteins; UA, uric acid; xCT, cystine/glutamate antiporter.

**Table 2:** Affinity of purine derivatives at the targets.

Purine Derivatives	Adenosine Receptor Subtypes				References
	A_1_AR	A_2A_AR	A_2B_AR	A_3_AR	
Antagonist potency for AR (*K_b_*, µM)					[62]
Caffeine	33.8	12.3	15.5	>100	
Paraxanthine	15.8	5.3	5.5	>100	
Theophylline	8.9	7.9	4.8	>100	
Agonist potency for AR (*EC*_50_, µM)					[62]
Adenosine	0.31	0.73	23.5	0.29	
Inosine	290	inactive	inactive	0.25	
Antagonist potency for AR (*K_i_*, µM) in striatum					[65]
Caffeine	20.5	8.6			
Paraxanthine	5.0	7.5			
Theophylline	4.7	9.8			
Theobromine	96.5	109.5			
Antagonist potency for AR (*Ki*, µM)					[66]
Theophylline	6.8	1.7	7.9	86	
Agonist potency for AR * (*IC*_50_, µM)					[67]
Adenosine	0.070	0.150	15.4	6.5	
Inhibition of AR (*IC*_50_, µM)					[68]
UA	>5300				
Caffeine	107				
	**Other Targets**				**References**
	**PDE**		**RyR**		
Inhibition of AR (*IC*_50_, µM)					[69]
Caffeine	400				
Caffeine			2000		

* Affinity for AR was analyzed by adenylate cyclase inhibition. Abbreviations: A_1_AR, adenosine A_1_ receptor; A_2A_AR, adenosine A_2A_ receptor; A_2B_AR, adenosine A_2B_ receptor; A_3_AR, adenosine A_3_ receptor, PDE, phosphodiesterase; RyR, ryanodine receptor.

**Table 3:** Neuroprotective effects of purine derivatives via regulating GSH synthesis and AR.

Treatment or Stimulus	Model	Analysis (Neuroprotective Activity)	Signal Transduction	References
UA	6-OHDA-treated PC12 cells (UA 200–400 µM)	LDH release↓, MDA↓, and 8-OHdG levels↓	SOD↑ and GSH↑	[133]
UA	6-OHDA-induced striatum lesioned rat (twice daily UA 200 mg/kg i.p. 10 days)	DA neuron loss↓, behavioral deficit↓, and MDA↓	SOD↑ and GSH↑	[8]
UA	6-OHDA-treated SH-SY5Y cells (200 µM UA)	cell viability↑	PI3K↑ and Akt/GSK3β↑	[8]
UA	MPTP treated Parkinson’s disease model mouse (UA 250 mg/kg i.p.)	Recovery of behavioral and cognitive function. DA neuron loss↓, GFAP^+^ astrocyte↓, and MDA↓	Nrf2 (nuc translocation)↑ GCLC/NQO1/HO-1↑, SOD↑, CAT↑, and GSH↑	[101]
UA	Cerebral ischemia/reperfusion model rat (UA 16 mg/kg i.v.)	TUNEL^+^ cell↓, MDA↓, carbonyl groups↓, and 8-OHdG levels↓	Nrf2/BDNF and NGF levels↑	[9]
UA	Mouse cortical astrocyte culture (100 µM UA)	Oxidative stress-induced DA neuronal cell death↓	Nuclear translocation of Nrf2↑, GCLM/NQO1/HO-1↑, and GSH synthesis and release↑	[37]
Caffeine	LPS-induced oxidative stress model mouse (Caffeine 30 mg/kg/day i.p. 4 weeks)	Apoptotic cell death↓ and synaptic dysfunction↓	Nrf2/HO-1↑ and TLR4/p-NF-κB/p-JNK↓	[134]
Caffeine	Cadmium-induced cognitive deficits model mouse (caffeine 30 mg/kg i.p. 2 weeks)	Neuronal cell loss↓ and synaptic dysfunction↓	Nrf2/HO-1↑	[135]
Caffeine	HT-22 and BV-2 cells (100 µM caffeine)	ROS↓ and lipid peroxidation↓	Nrf2/HO-1↑	[135]
Caffeine and UA	SIN-1 treated mouse (caffeine 10 mg/kg i.p. and UA10 mg/kg i.p.).	Nitrotyrosine levels in hippocampal slice↓	Cys uptake via EAAC1↑ GSH↑	[20]
Paraxanthine	H_2_O_2_ treated SH-SY5Y cells (10–100 µM paraxantine)	LDH release↓	Cys uptake via EAAC1↑ GSH↑	[21]
Guanosine	C6 astroglia and adult rat astrocyte culture (100 µM guanosine)	Azide-induced cytotoxicity↓	HO-1↑, GS/GR/GCL↑ and GSH↑	[136,137]
Guanosine	Cortical astrocyte culture (10 µM guanosine)	Oxygen/glucose deprivation and reoxygenation-induced cell death↓	A_1_AR agonism and A_2A_R antagonism, PI3K/Akt↑, and MEK/ERK↑	[138]
Guanosine	Rat hippocampal slices (100 µM guanosine)	Glu-induced cell death↓	PI3K/Akt//GSK3↑	[139]
Adenosine	Cerebral ischemia/reperfusion model rat (treatment with adenosine kinase inhibitor)	Infarct volume↓	A_1_AR agonism	[2,140]
Caffeine	MPTP treated mice (Caffeine 10–20 mg/kg, i.p.)	Striatal DA depletion↓	A_2A_AR antagonism	[5]
Caffeine, paraxanthine, and theophylline	MPTP treated mice (caffeine 10 mg/kg, Paraxanthine 10–30 mg/kg, Theophylline 10–20 mg/kg, i.p.)	Striatal DA depletion↓		[6]
Theobromine	Fat-enriched diet-induced cognitive deficits model rat (Theobromine 30 mg/L in drinking water)	Improve cognitive functions and Aβ and IL-1β levels in brain↓	A_1_R mRNA and protein level↑	[7]
Paraxanthine	MPP^+^ treated rat DA neuron culture (800 µM paraxanthine)	DA neuron loss↓	Ryanodine receptor activation	[84]

Abbreviations: Akt, protein kinase B; Aβ, amyloid-beta protein; BDNF, brain-derived neurotrophic factor; CAT, catalase; DA, dopamine; ERK, extracellular-signal regulated kinase; GCLC, GCL catalytic subunit; GCLM, GCL modifier subunit; GSK3, glycogen synthase kinase 3; HO-1, hemeoxygenase-1; IL-1β, interleukin-1β; i.v., intravenously injection; LDH, lactate dehydrogenase; LPS, lipopolysaccharide; MDA, Malondialdehyde; MPP^+^, 1-methyl-4-phenylpyridinium; MPTP, 1-methyl-4-phenyl-1,2,3,6-tetrahydropyridine, NGF, nerve growth factor; Nrf2, nuclear factor erythroid-2-related factor 2; NQO1, NAD(P)H quinone oxidoreductase 1; p-JNK, phosphor-c-Jun n-terminal kinase; PI3K, Phosphoinositide 3-Kinase; p-NF-κB, phosphor-NF-κB; ROS, reactive oxygen species; SIN-1, 3-morpholinosydnonimine; SOD, superoxide dismutase; TLR4, toll-like receptor 4,; TUNEL, Terminal deoxynucleotidyl transferase-mediated dNTP nick end labeling; 6-OHDA, 6-hydroxydopamine; 8-OHdG, 8-hydroxyl-2′-deoxyguanosine. Cell lines: BV-2, murine microglia cell line; HT22 cells, mouse embryonic fibroblasts; PC12 cells, rat pheochromocytoma cell line; SH-SY5Y cells, human neuroblastoma. ↑ and ↓ indicate an increase and decrease of the object, respectively.

## Abbreviations

5′NT, CD73	5′-nucleotidase
6-OHDA	6-hydroxydopamine
8-OHdG	8-hydroxyl-2′-deoxyguanosine
A_1_AR	adenosine A_1_ receptor
A_2A_AR	adenosine A_2A_ receptor
A_2B_AR	adenosine A_2B_ receptor
A_3A_R	adenosine A_3_ receptor
AD	Alzheimer disease
ADA	adenosine deaminase
Akt	protein kinase B
ApN	aminopeptidase N
Aβ	amyloid-beta protein
BBB	blood–brain barrier
BDNF	brain-derived neurotrophic factor
cAMP	cyclic AMP
CAT	catalase
CNS	central nervous system
CYP	cytochrome P450.
CYP1A2	cytochrome P450 family 1 subfamily A member 2
Cys	Cysteine
CysCys	Cystine
CysGly	Cysteinylglycine
DA	Dopamine
EAAC1	Excitatory amino acid carrier protein 1
ERK	Extracellular-signal regulated kinase
GCL	γ-glutamyl cysteine ligase
GCLC	GCL catalytic subunit
GCLM	GCL modifier subunit
GDA	Guanine deaminase
GLAST	Glutamate-aspartate transporter
Gln	Glutamine
GLT-1	Glutamate transporter 1
Glu	Glutamate
GluCys	glutamylcysteine
GluR	glutamate transporter
Gly	glycine
GPx	glutathione peroxidase
GR	GSH reductase
GS	glutamine synthetase
GSH	glutathione
GSK3	glycogen synthase kinase 3
GSK3β	glycogen synthase kinase 3 beta
GSS	GSH synthetase
GSSG	glutathione disulfide
GST	glutathione S-transferase
HO-1	heme oxygenase-1
IL-1β	interleukin-1β
i.v.	intravenously injection
Kir 4.1	Kir 4.1 potassium channel
LDH	lactate dehydrogenase
LPS	lipopolysaccharide
MDA	malondialdehyde
MEK	extracellular-signal regulated kinase kinase
MPP^+^	1-methyl-4-phenylpyridinium
MPTP	1-methyl-4-phenyl-1,2,3,6-tetrahydropyridine
MRP1	multidrug resistance protein 1
MRPs	multidrug resistance proteins
MX	methylxanthine
NF-κB	nuclear factor kappa-light-chain-enhancer of activated B cells
NGF	nerve growth factor
NQO1	NAD(P)H quinone oxidoreductase 1
Nrf2	nuclear factor erythroid-2-related factor 2
NTPDases, CD39	nucleoside triphosphate diphospho-hydrolase
PD	Parkinson’s disease
PI3K	Phosphoinositide 3-Kinase
p-JNK	phospho-c-Jun n-terminal kinase
p-NF-κB	phosphor-NF-κB
PNP	purine nucleoside phosphorylase
PX	paraxanthine
ROS	reactive oxygen species
SIN-1	3-morpholinosydnonimine
SOD	superoxide dismutase
TB	theobromine
t-BHQ	tert-butylhydroquinone
TP	theophylline
TUNEL	terminal deoxynucleotidyl transferase-mediated dNTP nick end labeling
UA	uric acid
xCT	cystine/glutamate antiporter
XO	xanthine oxidase
γ-GT	γ-glutamyl transpeptidase
